# Supplementary figures and images for: Integrated transcriptome and metabolome analyses revealed regulatory mechanisms of flavonoid biosynthesis in Radix Ardisia
Source: PeerJ. 2022 Jun 29;10:e13670. doi: 10.7717/peerj.13670 (PMC9250311; doi:10.7717/peerj.13670)

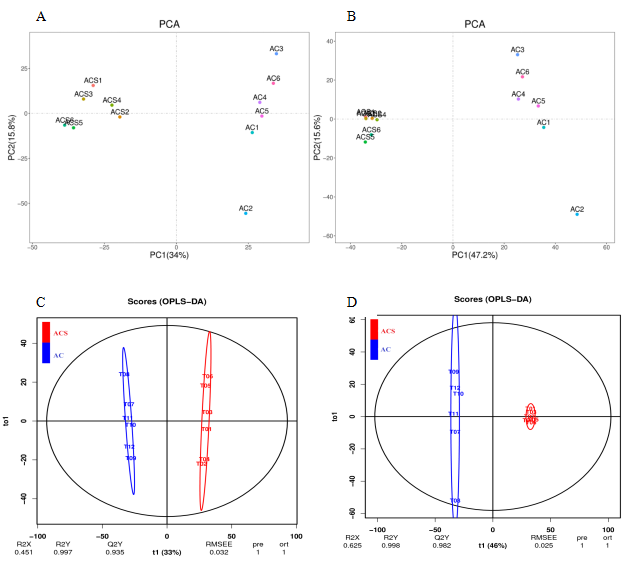

Supplement: Supplemental Information 1 [file peerj-10-13670-s001.png]

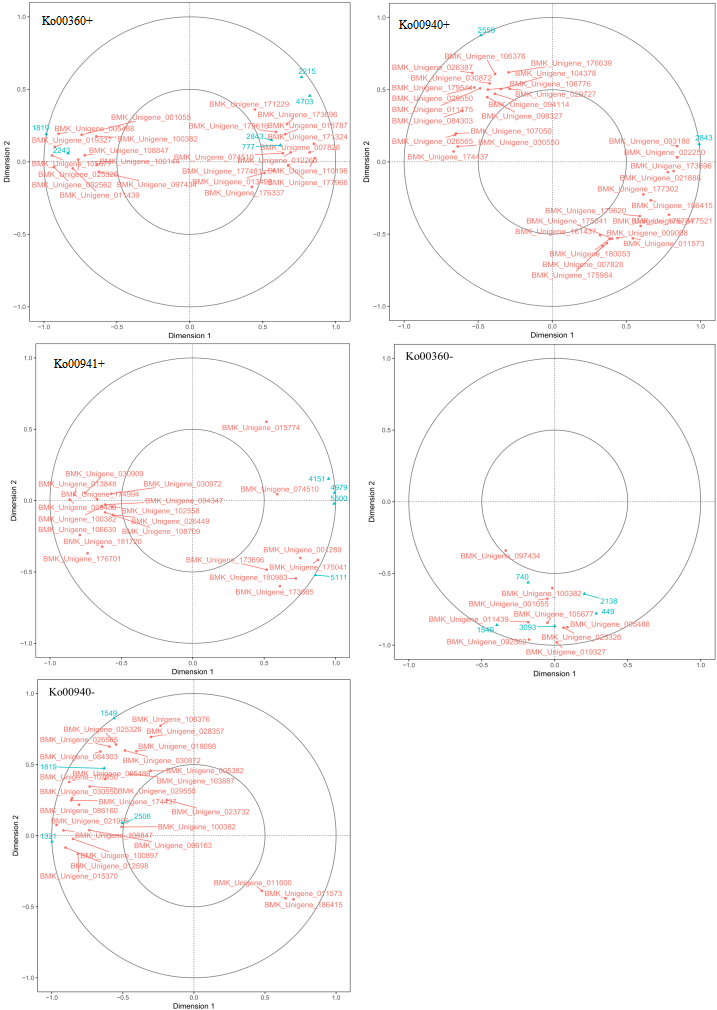

Supplement: Supplemental Information 2 [file peerj-10-13670-s002.png]

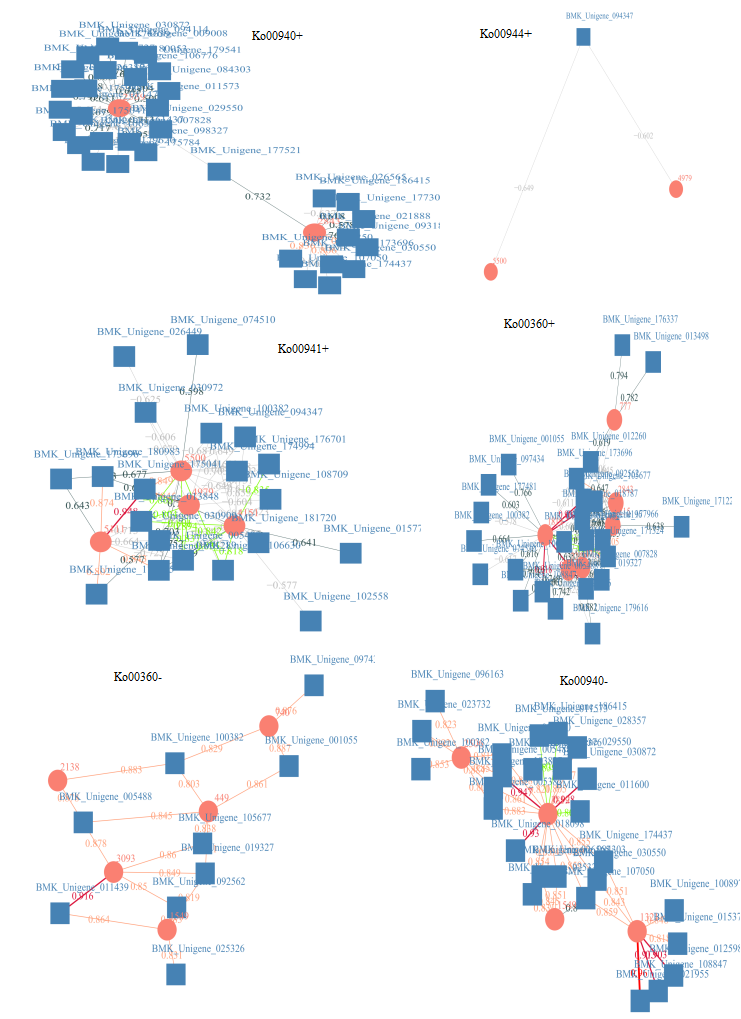

Supplement: Supplemental Information 3 [file peerj-10-13670-s003.png]
